# Genetic variants affecting equivalent protein family positions reflect human diversity

**DOI:** 10.1038/s41598-017-12971-7

**Published:** 2017-10-06

**Authors:** Francesco Raimondi, Matthew J. Betts, Qianhao Lu, Asuka Inoue, J. Silvio Gutkind, Robert B. Russell

**Affiliations:** 1 CellNetworks, Bioquant, Heidelberg University, Im Neuenheimer Feld 267, 69120 Heidelberg, Germany; 2 Biochemie Zentrum Heidelberg (BZH), Heidelberg University, Im Neuenheimer Feld 328, 69120 Heidelberg, Germany; 3 Graduate School of Pharmaceutical Science, Tohoku University, Sendai, Miyagi Japan; 4 Japan Science and Technology Agency (JST), Precursory Research for Embryonic Science and Technology (PRESTO), Kawaguchi, Saitama, Japan; 5 Moores Cancer Center, University of San Diego, San Diego, USA

## Abstract

Members of diverse protein families often perform overlapping or redundant functions meaning that different variations within them could reflect differences between individual organisms. We investigated likely functional positions within aligned protein families that contained a significant enrichment of nonsynonymous variants in genomes of healthy individuals. We identified more than a thousand enriched positions across hundreds of family alignments with roles indicative of mammalian individuality, including sensory perception and the immune system. The most significant position is the Arginine from the Olfactory receptor “DRY” motif, which has more variants in healthy individuals than all other positions in the proteome. Odorant binding data suggests that these variants lead to receptor inactivity, and they are mostly mutually exclusive with other loss-of-function (stop/frameshift) variants. Some DRY Arginine variants correlate with smell preferences in sub-populations and all 2,504 humans studied contain a unique spectrum of active and inactive receptors. The many other variant enriched positions, across hundreds of other families might also provide insights into individual differences.

## Introduction

Genomes from healthy individuals^1^ illuminate both diseases^2^ and attributes of individuals or human populations^3,4^. Perhaps the most common current use of these genomes is to provide background mutation rates to assess candidate disease mutations or to identify likely deleterious genetic alterations^5,6^. For example, ExAC^6^ is frequently used by human geneticists to determine variant frequency during searches for disease causing mutations. Studies of these data have uncovered sub-population differences between single nucleotide variants from healthy individuals in terms of affected pathways^7^ and between neutral, Mendelian and cancer variants^8^. However, perhaps owing to a view that variants in healthy individuals are not usually critical in a functional sense, there have been few studies into their consequences as might be uncovered by interrogation of protein structure/function (e.g.^9,10^) or by the analysis of conservation across close or distant sequence relatives (e.g.^11^). In essence: one does not usually explore in depth whether non-disease causing genetic variants are deleterious.

Protein coding genetic variants in a single gene can be important determinants of individual differentiation or disease. However, the complexity of biological function or disease often means that multiple genes and/or variants are required for a complete understanding or diagnosis. Sometimes mutations at equivalent positions in homologous proteins (i.e. as revealed by alignments and/or analysis of three-dimensional structures) can lead to similar diseases, such as K-, N-, H-Ras or kinase activating mutations at equivalent structural positions found in many cancers. A recent survey of cancer variants found several additional evolutionary or structurally equivalent protein positions affected by multiple cancers^12^. However, to our knowledge, a systematic search for structurally equivalent positions affected by variants seen in healthy individuals (and not attributed to diseases) has to date not been performed. Here we looked for instances of this phenomenon in thousands of genomes from healthy individuals mapped onto the set of known protein families.

Among the several families enriched in natural variants, we found, as expected, Olfactory Receptors (ORs) to be one of the most variant enriched families. The genetic variability of ORs is associated with a diverse functional repertoire and mammalian evolution^13^. There have been multiple studies to understand the functional consequences of mutations on odorant binding and signalling for certain receptors^14–17^. More recent investigations have begun to uncover determinants of odorant responsiveness and activation mechanisms^18,19^, highlighting the involvement of conserved residues, some of which we found to be significantly enriched in mutations in healthy individuals.

## Results

### Variant enriched protein families and positions

**Figure 1:**
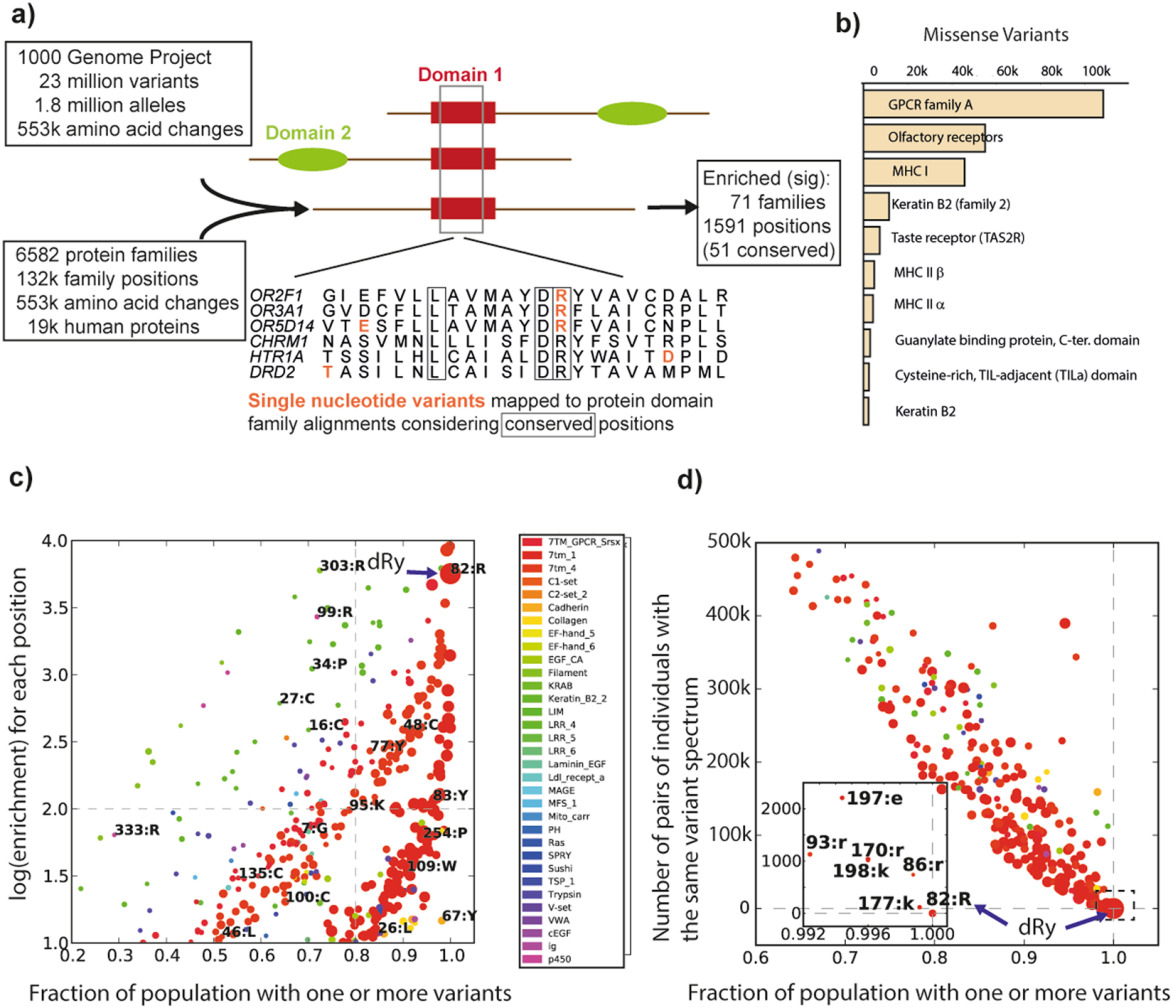
(**a**) Schematic showing how variant data are combined with aligned protein domains (coloured shapes; top), to identify equivalent alignment positions (below; boxes show conserved) and variants (red). (**b**) Protein domains having the most missense variants within the 1000 genomes population. (**c**) Enrichment at each significantly enriched alignment position (Q < = 0.01, ≥10 members with variants, log-odds > = 1) vs. the fraction of the total 1000 genomes population having at least one of these variants. Labels give Pfam alignment position and the most common residue (uppercase = conserved); colours denote Pfam families (7tm_1 and 7tm_4 are GPCR family A containing the DRY motif); diameter is proportional to variant count. (**d**) as for **c**) but where the y-axis is the number of times pairs of individuals have the identical spectrum of variants at a protein family position (i.e. dRy is the only position where no two individuals have the same spectrum, hence a value of zero).

We considered 23 million alleles from 2,504 individuals in the 1000 genomes dataset^20^, corresponding to 1.8 million unique missense variants, mapped to 553k unique protein changes (Fig. 1a). The 71 protein domain families significantly enriched are involved in functions indicative of individuality, including skin, hair, the immune system and sensory perception (Figs 1b and S1). Class A G-protein coupled receptors (GPCRs) have the most variants (Table S1) and the majority of these are within the Olfactory Receptor (OR) sub-family, known to contain many variants (e.g.^13,21^). We took care to avoid the inclusion of pseudogenes by considering only annotated human proteins, and inspection of family members, with previously published studies on pseudogenes (e.g.^22^) gave us confidence that we were only considering expressed, protein-coding genes. For ORs we specifically also considered the HORDE database, which both classifies known pseudogenes and marks several new candidates. We excluded all labelled pseudogenes, and considered any predicted pseudogenes in the sections that follow (see Table S2).

The 6582 protein family alignments had a total of 132k alignment positions (Fig. 1a), out of which just 1591 positions (1.2%) from 379 families (5.8%) are significantly enriched in variants (Table S3a), including 51 conserved positions (from 32 families, Table S3b). Some families, including GPCR, Keratin, Filament, Trypsin and EGF-like domains have positions showing both high variant enrichment and a high fraction of individuals with at least one variant (Fig. 1c). Most large families (e.g. kinases, Ras GTPases) have multiple conserved positions, but lack any with significant variant enrichment.

### Variants are highly enriched in the Olfactory receptor DRY motif

**Figure 2:**
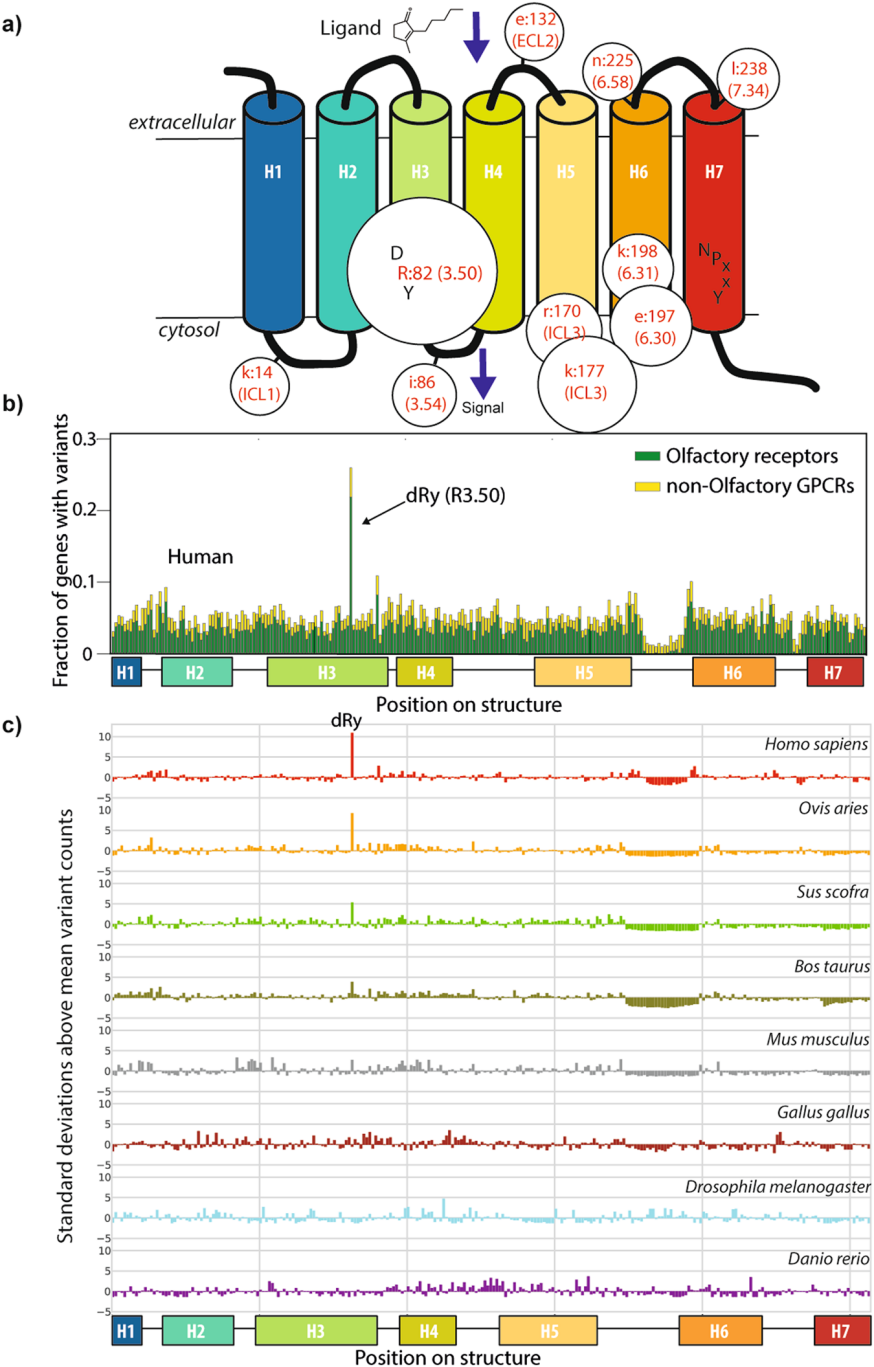
(**a**) Cartoon showing GPCR positions with the most variants; size is proportional to variant count, numbers denote Pfam 7tm_1 alignment positions, with the Ballesteros/Weinstein scheme given in parantheses. (**b**) Fraction of GPCR genes containing variants at each domain position showing the proportion in OR (yellow) and non-OR (green) receptors highlighted. (**c**) Plots of the number of the standard deviations above (positive) or below (negative) the mean for each position within GPCR family A for eight species within Ensembl variations^56^ having sufficient data for the analysis. The mean and standard-deviation are specific for each species and considering only variants within GPCR family A. The dip in the plot (between H5 and H6) is due to gaps within the alignment (i.e. fewer data points overall within this region).

Just one position in the proteome is highly conserved and significantly enriched (Q-value < 10^−300^), in variants that cover the entire population (i.e. each genome sequenced has at least one such variant): the Arginine from the DRY motif (dRy, R3.50 in Ballesteros/Weinstein, BW, numbering scheme^23^) of GPCRs, and the vast majority of these are in ORs. This position also stands out when considering how well positions define individuals (Fig. 1d): it is the only position in the proteome where no two of the 2,504 genomes have the same set of variants. There are several other significant positions, albeit with many fewer variants, on the cytosolic side structure (Figs 2a and S2b; including the “ionic-lock” residue at 6.30), suggesting a greater preference for variants affecting receptor activity and/or G-protein coupling compared to those affecting ligand binding.

The DRY motif, which lies near the cytoplasmic side of the third trans-membrane helix (TM3), is one of only two features conserved across most of the GPCR family^24^, the other being NPxxY towards the C-terminus of the protein (Figs 2a and S2b). It acts as a gate for signalling^25^ whereby ligand binding induces a conformational change that breaks a double salt bridge with an adjacent Aspartate/Glutamate residue (3.49) and with a charged residue on TM6 (6.30)^26^. The disruption of the ionic-lock and loosening of the constraints between TM3 and TM6 is linked to G-alpha subunit recognition and activation^27^.

Most dRy mutations have drastic effects on function, including some that lead to constitutive signalling^28^ and many others that alter or abolish it^25^. Few specific signalling experiments have been performed on ORs though inspection of a large OR/odorant binding screen^17^, suggests that dRy variants will likely disrupt function. Although only one of our observed specific variants was screened, the panel contains 24 ORs that lack the DRY Arginine in their canonical wild-type sequence (i.e. where the non-Arginine form is the major allele). Only one of the 1089 ligand/receptor screens for these dRy variants was positive, in contrast to 102 of 8815 screens for sequences having this Arginine, which is a greater than 12-fold enrichment and highly significant (hypergeometric p-value = 8 × 10^−5^; Table S4). Moreover, none of the other 15 conserved GPCR positions have anywhere near this degree of predictive power for receptor activation (Table S4). It should be emphasised that these experiments were not sufficiently detailed to determine whether functional variation was caused by defects in cell-surface expression^17^. Moreover, some receptors lacking the arginine appear to signal in non-canonical fashion (e.g. OR1G1^29^), which might not be detected in the luciferase assays used above.

We thus believe that dRy variants in ORs are likely to disrupt, or alter, function drastically. Studies of other, non-OR, GPCR R3.50 mutations^25,30^ suggest that this is likely due to a defect or alteration in G-protein signalling. It is well known that several ORs are segregating pseudogenes^17^, and we believe that ORs with dRy variants are also in this class. Indeed, 28 ORs where the canonical sequences have dRy variants are already predicted to be pseudogenes in HORDE^22^ (Table S2), likely, in part, because of the loss of this conserved residue.

**Figure 3:**
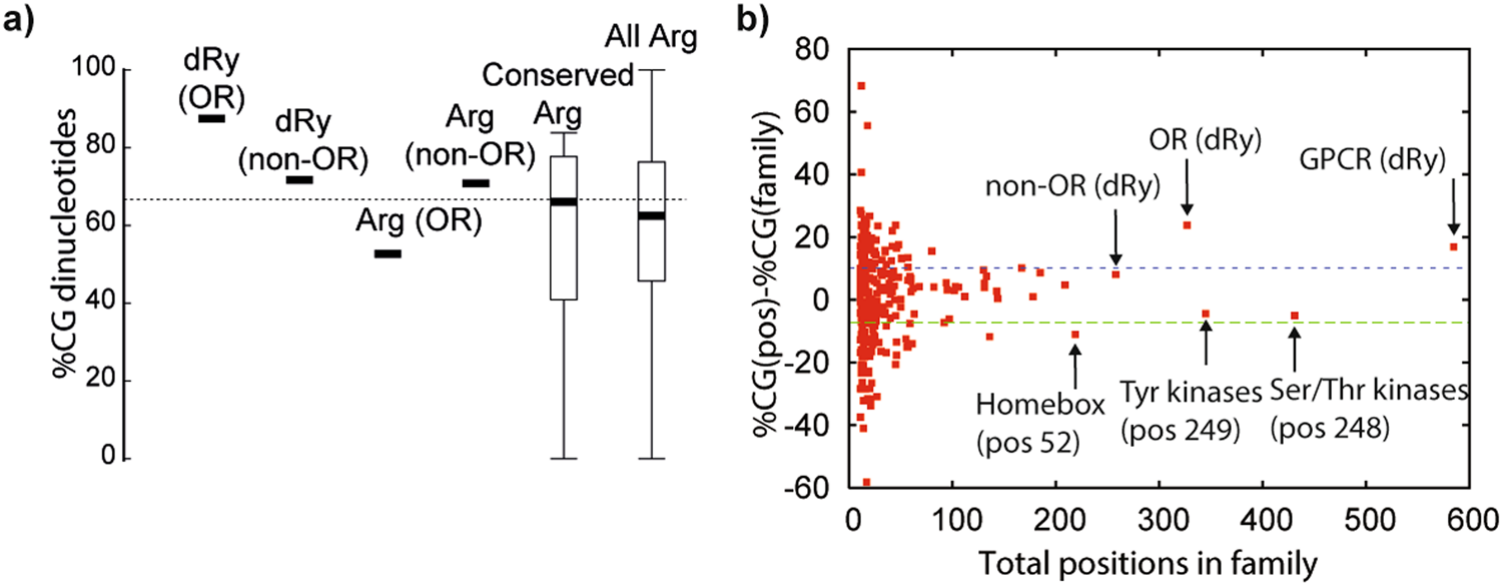
(**a**) Percentages of CG dinucleotides for different sets of Arginines; candlestick plots are shown where distributions are available. (**b**) The difference between the %CG for specific conserved Arginines and that for all domain family Arginines; key positions labelled by domain name and alignment position. Dashed lines show+/−1 standard deviation for positions in domains with at least 20 sequences.

The dRy position has the highest variant number and more genes with variants than any other GCPR position (Figs 2b and S2) and the vast majority of variants (85.9% of variants, 99.6% of alleles) are from ORs. All 37 non-OR dRy variants are rare (<0.5% minor allele frequency) and heterozygous; in contrast several ORs variants are very common (Table S5a,b). Despite a generally increased overall variant count in ORs, dRy still stands out when compared to other Arginines (Table S6a,b), including others within ORs. We also observe a similar phenomenon in other mammals, though not in other vertebrate or metazoan species (Fig. 2c). Arginines are more prone to mutations owing to CG dinucleotides^8^; interestingly OR dRy is more enriched in CG nucleotides than other Arginines within ORs or within the wider proteome (Fig. 3).

Given the inherent mutability at this position, a logical question is whether there is any evidence of positive selection involving these variants. To test this, we built trees^31^ using alignments of 5008 sequences (two per genome) for each OR and then used PAML^32^ to test for positive selection of both genes and individual amino acid positions (Table S7). Considering the 52 genes for which there are at least two distinct dRy variant sequences (i.e. where informative trees can be made involving this position), 32 of these show grouping of dRy variants in a single clade, 21 of which have high posterior probabilities (> = 0.9) for the separating branches, and 18 have high PAML positional positive selection probabilities (> = 0.9). Being more restrictive by requiring five sequences (i.e. better trees) improves this considerably: 15 of 17 sequences show grouping with 9 and 10 having high posterior or PAML probabilities. Examples of these trees are given in Figs S3 and S4.

It should be noted that there are several other, non dRy, positions that also show similar evidence for selection, and many of these are indeed other CpG codon Arginines. However, there is still a slight, though not significant, enrichment of dRy CG codon variants over the others (1.35 fold). Overall, this analysis suggests some evidence for positive selection at this position, though it remains a possibility that these dRy variants are the result of negative selection acting on a CG-rich Arginine codon. Many studies have suggested a relaxed negative selection in ORs within humans (e.g.^32–34^). However, we believe that our observations, together with what is known of the function of this residue in GPCRs, support a functional consequence for these variants.

Humans have, on average, 9.3 OR dRy variants spanning 186 receptors and are usually heterozygous for these alleles (6.8 heterozygous and 1.3 homozygous variants on average; Fig. S5, Table S5a); dRy variants in non-ORs are all heterozygous (Table S5b). As expected from the higher mutation rate and the high number of pseudogenes^35–37^, ORs also contain loss-of-function mutations: a total of 162 have stop-gains and 179 have frameshift variants, with 37 dRy, 29 stop and 72 frameshift mutations with allele frequencies of 1% or higher. Including dRy variants, 318 ORs have at least one likely loss-of-function variant, and on average, humans have 39.5 OR highly deleterious OR mutations (26.4 heterozygous and 6.5 homozygous). Interestingly, one of these three loss-of-function variant types typically predominates: for 112 of the 121 (93%) ORs having at least one dRy, stop-gain or frameshift with allele frequency > = 1%, one type accounts for the vast majority (> = 90% of the total; Table S8). Just 52 ORs lack any of these deleterious variants.

### dRy and loss-of-function variant segregation in human sub-populations

**Figure 4:**
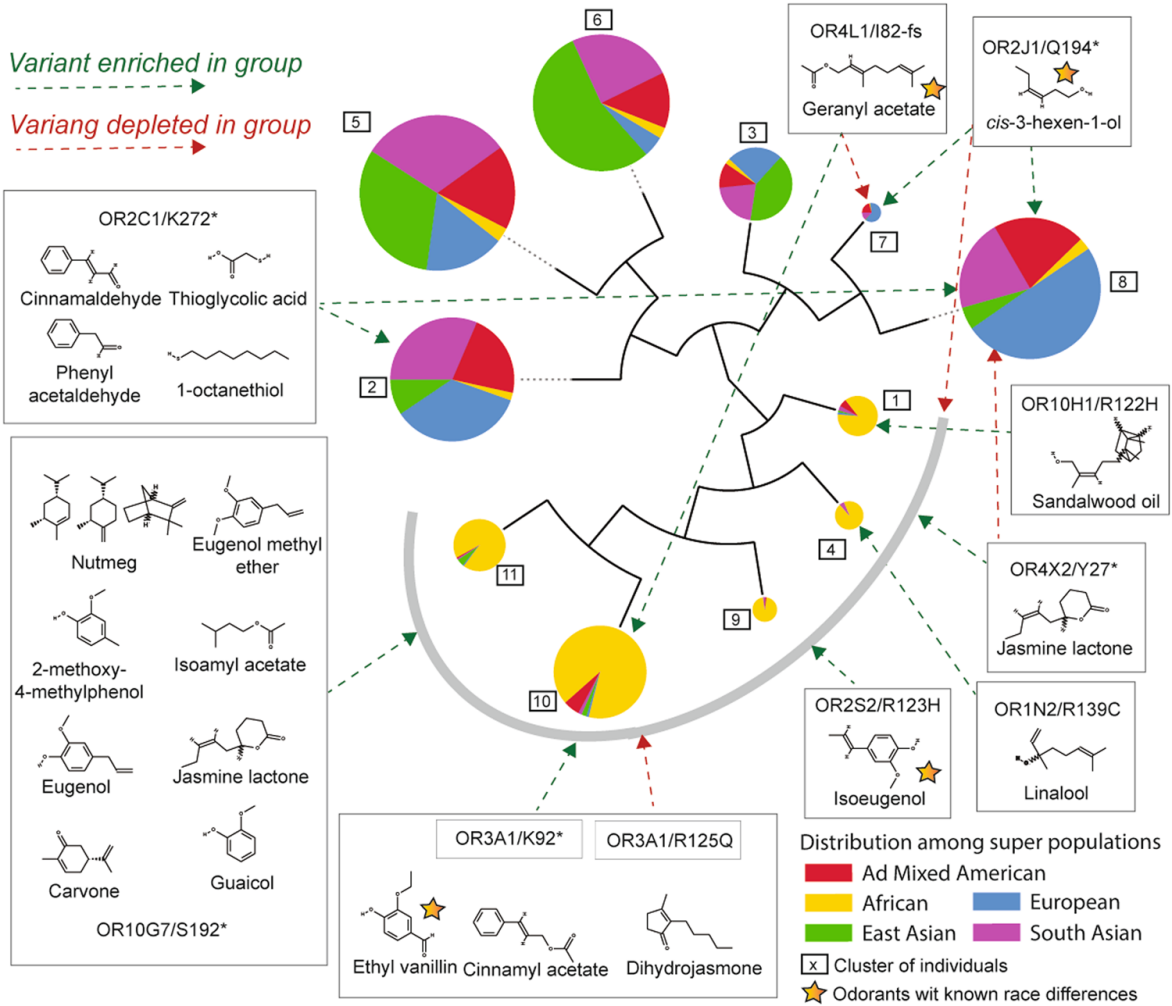
Hierarchical clustering dendrogram of 2,504 individuals based on OR loss-of-function (dRy, stop-gain or frameshift) fingerprint distances. Clustered pie charts showing the proportions of sub-populations containing the variants; radii are proportional to population; colours show 1000 Genome super-population composition. Loss-of-function variants are shown as positional changes given as wild-type residue, position and change, where * denotes stop-gains and fs denotes frameshifts (those labelled are dRy variants). Only variants enriched/depleted (log odds ≥ 0.6 or ≤ −0.6) in one 1000 Genomes super-population relative to the others are shown. Variants are shown in boxes together with known ligands for the receptors^17^ and arrows show if they are enriched (green) or depleted (red) in a particular super-population. The grey curve around the mostly African (yellow) clusters is for clarity; arrows touching this line indicate enrichment or depletion in all clusters.

Clustering according to the OR dRy and loss-of -function variant spectrum (i.e. the set of variants present in each individual) shows a reasonable division into human sub-populations (Fig. 4). As expected by the Out-of-Africa hypothesis, African dominated clusters show the greatest number of variants, including many in OR dRy (Fig. 4, Table S9). This is in broad agreement with a previously observed lower average olfactory acuity in people of African descent^38^.

Several dRy or loss-of-function variants show limited correlation with specific olfactory differences between human populations (Fig. 4; Tables S9 and 10). Only eight odorants with observed significant differences in perception across individuals of African, Asian or European ancestry^38^ are known to bind specific receptors^17^. Though many odorants bind multiple receptors, and how odorants ultimately relate to odor preferences is complicated by many factors (genetic or otherwise), we find loss-of-function variants that might help explain aspects of this complex relationship. We do not suggest that these variants will fully explain responses to specific odorants or that there are not variants other than dRy or loss-of-function that correlate with olfaction differences. Rather, we are simply presenting correlations between dRy/loss-of-function variant enrichment and published olfaction differences that might help illuminate this complex process. Variants leading to major functional differences are attractive experimentally as they are more likely to lead to a phenotype than those that potentially have more subtl e functions (such as differences in ligand affinity).

In OR3A1, for example, dRy or stop-gain (at position K92) variants (considered together) are half as common in Africans compared to Europeans, and these groups differ in their response to the ligand ethyl vanillin. Differences in geranyl acetate perception between Asians and Europeans is correlated with greater frequency of either OR4L1 frameshift or OR2J3 dRy variants. The greater sensitivity to isoeugenol in Europeans as compared to Africans or Asians correlates with a near complete lack in Europeans of several stop-gain or dRy variants in the isoeugenol binding receptors OR2J2, OR10A6 and OR2S2 that are seen in Africans and Asians (Tables S9 and S10). A common stop-gain in OR2J1, with a very high frequency in Europeans, but lower in the other groups also correlates with olfaction differences regarding its ligand cis-3-hexen-1-ol, though these are not significant^38^. Differences in the perception of this odorant have been linked to non dRy or loss-of-function variants in OR2J3^39^, which is an unusually close homolog of OR2J1 (88% sequence identical; a likely recent paralogue and likely to have a very similar function). There are also several variants that are highly enriched in sub-populations, but which lie in receptors currently lacking any information about ligands. For instance, OR2L2/R121H in Africans, OR2T7/R113H in South Asians and OR13A1/R140Q in Americans and OR4X1/R120C in Europeans.

These genetic differences are not perfect discriminators of odorant responses in human sub-populations, but the high frequency of many might help account for average differences in olfaction (i.e. over hundreds of individuals). Moreover, we do not rule out that many other positions, apart from dRy, can also have drastic functional consequences and thus affect odorant response (e.g. variants in OR2J3 above).

By considering all odorants and all receptors where binding data are available^17^, possibly broader trends become apparent, including sub-population differences in response to dihydrojasmone, menthol & cinnamyl acetate (Fig. S6). For several individuals and several odorants, the predicted anosmia would possibly involve defects in more than one receptor binding that odorant (Table S11). For example, considering jasmine lactone, there are 27 individuals (all African) with loss-of-function variants in two receptors (OR10G7 and OR4X2) found to bind this odorant. More directed investigations, coupling olfaction studies with the genotyping of specific variants, would likely be greatly illuminating.

### dRy variants in non-Olfactory GPCRs

1000 genome dRy variants in non-Olfactory GPCRs are rare and always heterozygous (Table S5b), suggesting that they are not generally tolerated in healthy individuals. Indeed, several have been already linked to diseases, including P2Y12/R122C in bleeding^40^ and AVPR2/R137(C/L/H) in nephropathies^41^. Certain tumors also have mutations at this site in both Olfactory and non-Olfactory receptors^42^ suggesting that certain dRy mutations could also play roles in cancer.

### Other protein families with variant enriched positions

There are several other significant positions in other protein domain families (Table S3). Though these do not stand out to the same degree as the OR dRy variants, they nevertheless might represent other sites on the proteome where germline variability differentiates healthy individuals.

For example, a conserved position in Cadherin domains (Cadherin:67 in Table S3b) is significantly enriched in variants within a mostly Procadherin subgroup (25 of 30 genes with variants). This position is usually in a hydrophobic pocket close to the calcium binding site^43^. Mutations here would be predicted to have an effect on the stability on specific Cadherin repeats. Protocadherins lie at cell junctions, particularly in neurons, and the multiplicity of genes, splice variants and glycosylated forms is thought to lead to a great diversity of links between neurons^44^. Interestingly, recent studies have linked rare variants at (or immediately adjacent to) these positions to neurological disorders (e.g.^45^).

Elsewhere, variants are also enriched at a conserved Cytochrome p450 Arginine (p450:99 in Table S3b) involved in heme stabilization^46^. Variants at this site are known to alter either structure or metabolism in several p450s, including CYP3A4^47^ and CYP2A6^48^. The majority of the observed variants are within CYP2A7, which is a Leucine instead of Arginine in nearly half of all alleles, though there are rarer variants in another 12 of the 60 human enzymes. These variants (and several other p450 variants) provide additional candidates to probe xenobiotic metabolism differences.

## Discussion

The analysis of variants or mutations at structurally or evolutionarily equivalent positions in protein families has been illuminating for cancer^12^. We believe our results show this approach can also be equally informative for the study of individual variation within a species. The hundreds of positions identified are a starting point to probe genes that could determine subtle differences in human physiology and behavior. It is telling that the families identified are not among those normally considered critical for organism survival (e.g. kinases, GTPases), but are large families that appear to share the common features of redundancy and great functional diversity. A loss-of-function event in a single OR, or one Cadherin repeat within one of several multi-domain Cadherins, is unlikely to have the same effect as an equivalent event in a critically important protein like CDK2 or HRAS. The protein families that contain these equivalent variants appear either to be individually not essential (e.g. ORs), to occur naturally in slightly different states in different individuals (e.g. Cadherins), or to perform related, often overlapping functions (e.g. cytochromes p450).

The genetic variability of Olfactory receptors, including gain, loss and pseudogene formation, is associated with a diverse functional repertoire^13^. The system appears inherently redundant, with multiple odorants binding to the same receptor and vice versa, which means that the loss of one would most often be tolerated. OR genes are reduced in primates and it has been argued that the accumulation of deleterious mutations could further decrease olfactory capability in humans^49,50^. Loss or gain of signaling in dRy variants might be another means to diversify the olfactory responses and to provide related populations with a more similar odorant (and environmental) response.

The fact that common loss-of function, and thus likely binary (on/off), OR variants agree with some odor preferences raises the question whether differences in smell perception could be an important feature in mammalian populations. Offspring will share considerable odorant responses with parents, which might make them inclined to stay in a similar odor environment, though occasionally an individual might inherit a variant profile making the environment distasteful, prompting an exodus. Simple simulations support this (Suppl. Movie 1) and odorant driven movement leads to small, genetically homogeneous communities reminiscent of mammalian social organisation^51^ that occasionally spawn sub-colonies. Surprisingly, these simple simulations also suggest a greater genetic diversity in a smaller overall population (Fig. S7).

Overall, these natural variant enriched protein family positions offer considerable possibilities to understand human diversity. More generally, the systematic integration of high-throughput sequencing data and information regarding evolutionary conservation or molecular mechanism is a promising means to understand the complex relationship between genotype and phenotype.

## Materials and Methods

### Datasets

We downloaded missense variants from Phase III of the 1000 Genomes project^1^ and mapped them to Uniprot^52^ via the Variant Effect Predictor from Ensembl^53^. We identified Pfam-A^54^ families within the mapped Uniprot Swissprot sequences using HMMer^55^. We also considered SNP datasets from Ensembl Variations^56^ of the eight species having more than 6 million variants (*Homo sapiens, Mus musculus, Bos taurus, Sus scrofa, Ovis aries, Gallus gallus, Danio rerio, Drosophila melanogaster*). For the analysis of enriched variants, we did not consider indels or other variants as these are both fewer in number and often lead to uncertain consequences in terms of individual protein positions. We calculated the fraction of nonsynonymous/synonymous variants by considering the average minor allele counts for dRy variants, other Arginines or any position within OR and non-OR GPCRs (human variants only).

### Variant enrichment

To define positions within protein family alignments enriched in variants we computed the log odds as the log of the observed number of variants divided by the expected. We computed the expected number by multiplying the frequency of total alleles (>23 million) in the total proteome length (>11 million amino acids) times the number of domain instances in the proteome, times the domain length (Pfam-A model length). When evaluating the enrichment of individual domain positions, the expected number was corrected only for the number of domain instances in the proteome. We assessed the statistical significance of whole domain or domain position enrichments through a binomial test, computing the prior probability by randomly shuffling of missense protein variants from each individual across the same protein. We corrected P-values through the False Discovery Rate/Benjamini-Hochberg procedure to give a corresponding Q-value. We only considered positions having 50 observed or 5 expected missense variants and defined enriched positions as those with log odds > = 1 and Q < = 0.01.

We computed gene-ontology (GO) term enrichment (biological process & molecular function) as described previously (http://getgo.russelllab.org)^57^, considering genes from families significantly enriched in variants. To study codon usage we compared human cDNA sequences from Ensembl^53^ against the Uniprot/Swissprot sequences used above using BLASTX^58^ and considered best identical sequences matches. We defined conserved positions within protein families those with a strong HMMsearch^55^ alignment consensus.

### Receptor/odorant data analysis

We pooled systematic screening data from studies on ligands activating mouse and human Olfactory receptors^17,59,60^. We used the alignment to the Pfam 7tm_1 domain (as above) to determine whether screened receptors had canonical or non-canonical residues at the 16 highly conserved positions within this family (Pfam consensus). We computed the frequencies of positive tests, either by counting those passing the threshold, or by EC50 weighted counts computed as −1 * log(EC50)/9 (as −9 was the strongest log(EC50) value seen). We then divided the ratio of canonical/non-canonical receptors for each position, and the associated Hypergeometric probability of seeing this many or fewer positives (un-weighted values only) to give the values in Table S4.

To estimate potential anosmia arising from loss-of-function variants, we summed log(EC50) values, for each allele, in each of 2505 individuals, for all the odorant-receptor pairs, unless a the individual had a loss-of-function variant (either dRy, stop-gain or frameshift). We considered the overall response of an individual to a given odorant as the average of the log(EC50) values. To draw the matrix in Fig. S7, we considered the average of all the individual responses to a given odorant for the members of the same population group and we normalized each ligand response relative to the maximum value.

For the discussion of olfactory responses in human populations, we used data on olfaction demographics from a previous study^38^.

### Clustering

We clustered individuals based on the allelic variant profiles of selected SNPs by defining binary fingerprints of allelic variants, calculating distances between them as the sum of absolute differences between each fingerprint (scipy.org pdist function), and performed complete linkage, hierarchical clustering (scipy.org libraries). We estimated the number of identical individual matches on Fig. 1d through distance calculation based on fingerprints derived from domain position alleles. We constructed the tree (Fig. 4) using the ete library^61^ with a depth cut-offs (chosen by visual inspection) of 35 bits (for dRy) and 100 (all OR variants). We obtained associations of odorants/ligands to receptors from systematic investigations of olfactory receptor preferences (see above). We defined enriched (or depleted) variants (and associated ligands) as those having a log odds ratio between their frequency within the cluster and in the remaining of the population above or below a particular threshold (+/−0.6 in Fig. 4).

### Assessing evolutionary selection

For each gene we used the set of non-redundant transcript coding sequences to build trees with MrBayes^31^ under the standard model of nucleotide substitution, with different substitution rates for transitions and transversions, run on four chains for 1,000,000 generations to convergence (standard deviations of split frequencies in every case were < = 0.05), and sampled every 100^th^ generation of the final 750,000 to generate majority-rule consensus trees of all compatible partitions. We then used codeml from the PAML package^32^ to assess both overall selection pressure (single dN/dS ratio per tree) and different rates at different sites (three discrete site classes with different dN/dS ratios). We discarded trees as uninformative when the model of different rates at different sites had collapsed to the same rate for every site (likely indicating too little variation for meaningful phylogenies). We inspected and displayed the resulting trees using iTOL^62^.

### Simulations of odorant driven behavior

We simulated the growth, movement, mating & evolution of organisms in a 200 × 200 grid, where we placed 50 odorants randomly and 40 starting individuals close together initially. Each individual is given a set of 100 receptor genes, each of which have between one and 14 randomly selected odorant ligands, and which are set to drive attraction or repulsion with equal probability. Simulations involve each animal moving (to an unoccupied adjacent grid point) either randomly or according to the net attraction/repulsion vector,

$$\mathop{{m}_{a}}\limits^{\longrightarrow}=\sum _{i\,odorants}\sum _{j\,receptors}\frac{A{R}_{ij}}{{d}_{ia}}\frac{\,(\mathop{{o}_{i}}\limits^{\longrightarrow}-\,\mathop{{p}_{a}}\limits^{\longrightarrow})}{\Vert \,(\mathop{{o}_{i}}\limits^{\longrightarrow}-\,\mathop{{p}_{a}}\limits^{\longrightarrow})\Vert }$$

where $\mathop{{o}_{i}}\limits^{\longrightarrow}$ contains the coordinates of the odorant, $\,\mathop{{p}_{a}}\limits^{\longrightarrow}$ is the coordinates of the animal, *d*_*ia*_ is the distance between them and *AR*_*ij*_ is 1, −1 or 0 to denote attraction, repulsion or no response for each of *j* receptors in animal *i*. The unit vector corresponding to $\mathop{{m}_{a}}\limits^{\longrightarrow}$ is then used (i.e. to limit steps to be one grid point in magnitude).

Movements were limited to one grid point in the x and/or y direction according to this vector. Animals of opposite sex are permitted (30% probability) to mate if they are in adjacent cells and if there are at least five empty cells around one parent. Offspring have randomly selected genotypes taken equally from each parent, with a 5% chance of alleles also changing randomly (i.e. random mutations). We repeated several simulations for a common initial set of animals, genotypes and odorant positions varying the fraction of odorant driven moves (intervals between 0 and 100%).

## Electronic supplementary material

All supplementary text & figures together

All supplementary tables together (different tabs)

Movie S1
