# Supplementary material for: Genetic variants affecting equivalent protein family positions reflect human diversity
Source: Sci Rep. 2017 Oct 6;7:12771. doi: 10.1038/s41598-017-12971-7 (PMC5630595; doi:10.1038/s41598-017-12971-7)
Supplement: Supplementary file 1 — All supplementary text & figures together [file 41598_2017_12971_MOESM1_ESM.pdf]

Supplementary Information

Genetic variants affecting equivalent protein family positions reflect human diversity

Francesco Raimondi (1,2), Matthew J. Betts (1,2), Qianhao Lu (1,2), Asuka Inoue (3,4), J. Silvio Gutkind (5), Robert B. Russell* (1,2)

1. CellNetworks, Bioquant, Heidelberg University, Im Neuenheimer Feld 267, 69120 Heidelberg, Germany
2. Biochemie Zentrum Heidelberg (BZH), Heidelberg University, Im Neuenheimer Feld 328, 69120, Heidelberg, Germany
3. Graduate School of Pharmaceutical Science, Tohoku University, Sendai, Miyagi, Japan
4. Japan Science and Technology Agency (JST), Precursory Research for Embryonic Science and Technology (PRESTO), Kawaguchi, Saitama, Japan.
5. Moores Cancer Center, University of San Diego, San Diego, USA

* Corresponding author:

Email: bq_rrussell@bioquant.uni-heidelberg.de

Tel: +49 6221 54 51 362

Supplemental Figures & Tables

Figure S1

GO biological processes linked to genes in families most affected by variants; percentages of genes are shown in the chart or next to the names.

Figure S2

a) Allelic counts versus position within the structure **b)** cartoon of a GPCR representative structure (PDB code 4eiy) showing the top 10 positions most affected by variants, shown as spheres centered on Cβ or Cα atoms. The size of the spheres is proportional to the variant counts as for Figure 3a.

Figure S3

Tree of OR3A1 dRy alleles generated from the PAML analysis. The branches corresponding to dRy minor alleles are highlighted in orange.

Figure S4

Same representation as Fig S3 for OR8D2.

Figure S5

Hierarchical clustering of 2,504 individuals (Y-axis) based on distances between fingerprints of OR dRy alleles within ORs (X-axis). Darker red dots indicate homozygous variants. Left and bottom dendrograms indicate similarity of groups of individuals/receptors based on OR dRy fingerprints. Dendrogram color scheme reflect clustering based on fingerprint similarity. Left color band between dendrogram and main matrix indicate super population membership of each individual. The matrix at the top shows associations between odorants and olfactory receptors(35).

Figure S6

Anosmia estimation based on observed loss-of-function variants. For each allele in each of 2504 individuals, we summed log(EC50) values for all the odorant-receptor pairs, unless a deleterious variant (either dRy, Stop gain or Frameshift) was observed. We

considered the overall response of an individual to a given odorant as the average of the log(EC50) values. We considered the average of all the individual responses to a given odorant for the members of the same population and we normalized each ligand response relative to the maximum value.

Figure S7

a) Genotypic diversity versus the number of animals in 50 simulations (ten for each of 0, 25, 50, 75 or 100% odorant driven movement) for eight different random starting environments. Note how the higher odorant driven simulations (yellow, orange, red) tend to be towards the bottom of the plots indicating the highest diversity in genotype. **b)** Genotypic diversity versus the number of iterations (time step) in 50 simulations (ten for each of 0, 25, 50, 75 or 100% odorant driven movement) for eight different random starting environments. Note how the higher odorant driven simulations (yellow, orange, red) tend to be towards the bottom of the plots indicating the highest diversity in genotype.

Table S1

Protein family domains most enriched in missense variants.

Table S2

ORs from Swissprot/Uniprot annotated with family and pseudogene classification (from HORDE) and with deleterious mutations information (dRy, Stop gains and frameshifts)

Table S3

Protein family domain positions most enriched in missense variants.

Table S4

Assessing the ability of mutations from canonical GPCR residues to predict odorant binding from OR/odorant screens (19, 34, 35)

Table S5

dRy missense variants and associated Minor Allele Frequency (MAF) and zigosity for **a)** non-OR GPCRs and **b)** OR GPCRs.

Table S6

a) Total counts of variants and total number of positions for different Arginine positions within humans. Ratios (rightmost column) and enrichments (bottom) are also shown. **b)** Amino acids mutation enrichments and log odds in OR and non-OR genes relative to other genes. **c**) Non-synonymous/synonymous variants ratio for variants at dRy as well as other positions for OR/non-OR. **d**)Non-synonymous/synonymous variant ratio for all human genes .

Table S7

PAML analysis of OR alleles from the 1000 Genomes project

Table S8

dRy, stop-gain and frameshift variants within ORs .

Table S9

OR dRy arginine missense variant enrichment within each continental group.

Table S10

a) Enrichment of OR dRy variants within clusters of individuals defined through variant profile similarity and **b)** ethnic group composition of each cluster.

Table S11

Estimation of Anosmia by combining information on OR loss-of-function alleles in each of 2505 individuals in the 1000 genomes dataset with information on odorant/OR binding data taken from Mainland et al.

Movie S1

Examples of simulating animal behavior in a 2D matrix with scattered odorants and where animals contain 100 receptor genes that determine attraction or repulsion to one or more odorants. More details are given in the movie itself and in Online Methods.

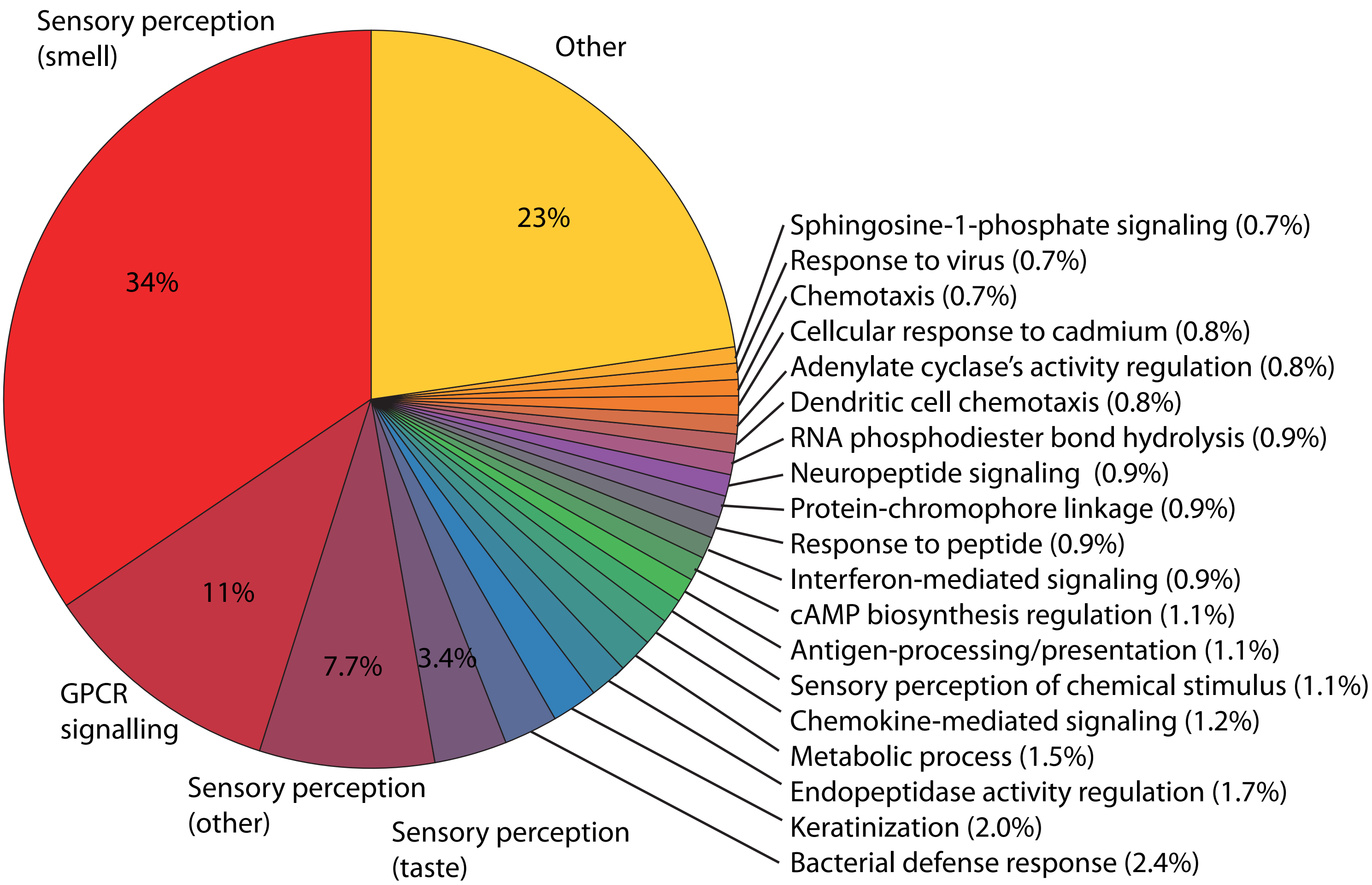
Sensory perception (smell)
34%
Other
23%
GPCR signalling
11%
Sensory perception (other)
7.7%
Sensory perception (taste)
3.4%
Sphingosine-1-phosphate signaling (0.7%)
Response to virus (0.7%)
Chemotaxis (0.7%)
Cellcular response to cadmium (0.8%)
Adenylate cyclase's activity regulation (0.8%)
Dendritic cell chemotaxis (0.8%)
RNA phosphodiester bond hydrolysis (0.9%)
Neuropeptide signaling (0.9%)
Protein-chromophore linkage (0.9%)
Response to peptide (0.9%)
Interferon-mediated signaling (0.9%)
cAMP biosynthesis regulation (1.1%)
Antigen-processing/presentation (1.1%)
Sensory perception of chemical stimulus (1.1%)
Chemokine-mediated signaling (1.2%)
Metabolic process (1.5%)
Endopeptidase activity regulation (1.7%)
Keratinization (2.0%)
Bacterial defense response (2.4%)

Figure S1

a)

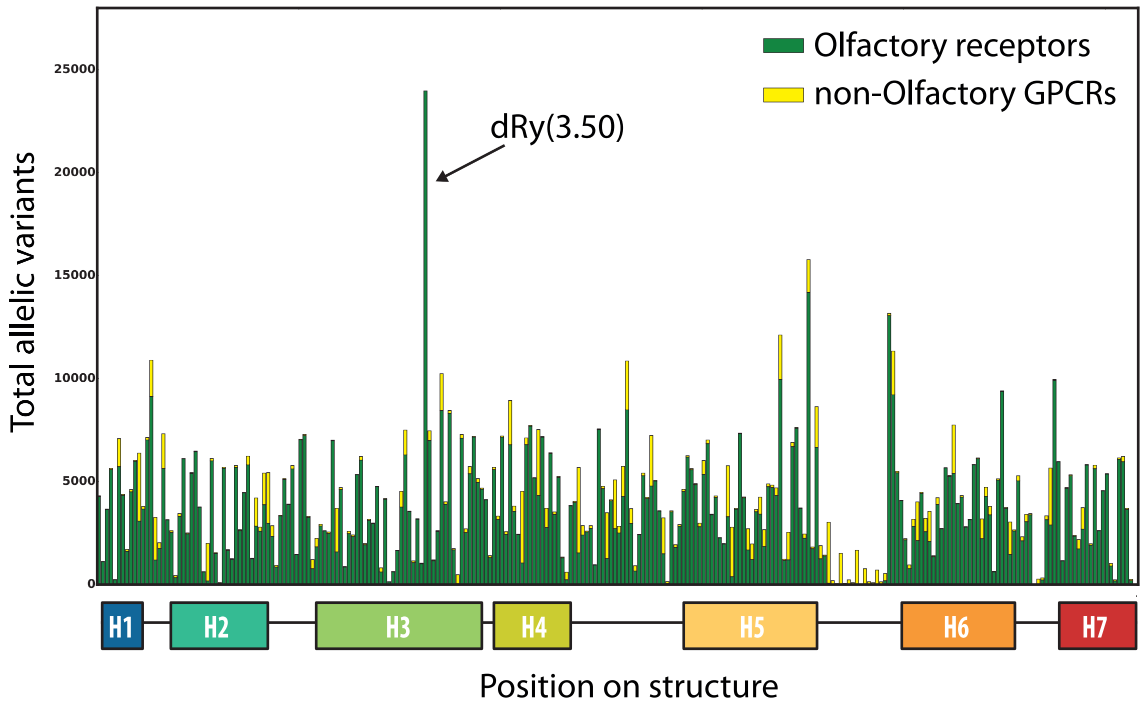

Olfactory receptors
non-Olfactory GPCRs
dRy(3.50)
Total allelic variants
25000
20000
15000
10000
5000
0
H1
H2
H3
H4
H5
H6
H7
Position on structure

b)

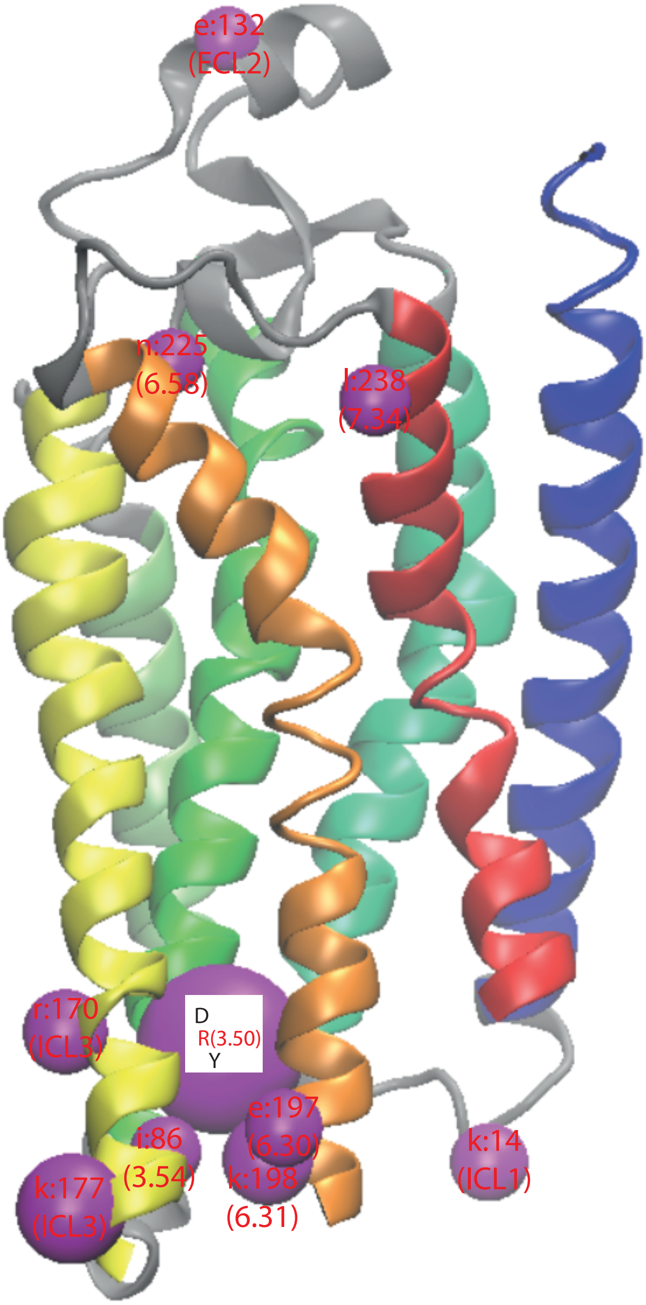

e:132 (ECL2)
n:225 (6.58)
l:238 (7.34)
r:170 (ICL3)
D R(3.50) Y
e:197 (6.30)
i:86 (3.54)
k:198 (6.31)
k:14 (ICL1)
k:177 (ICL3)

Figure S2

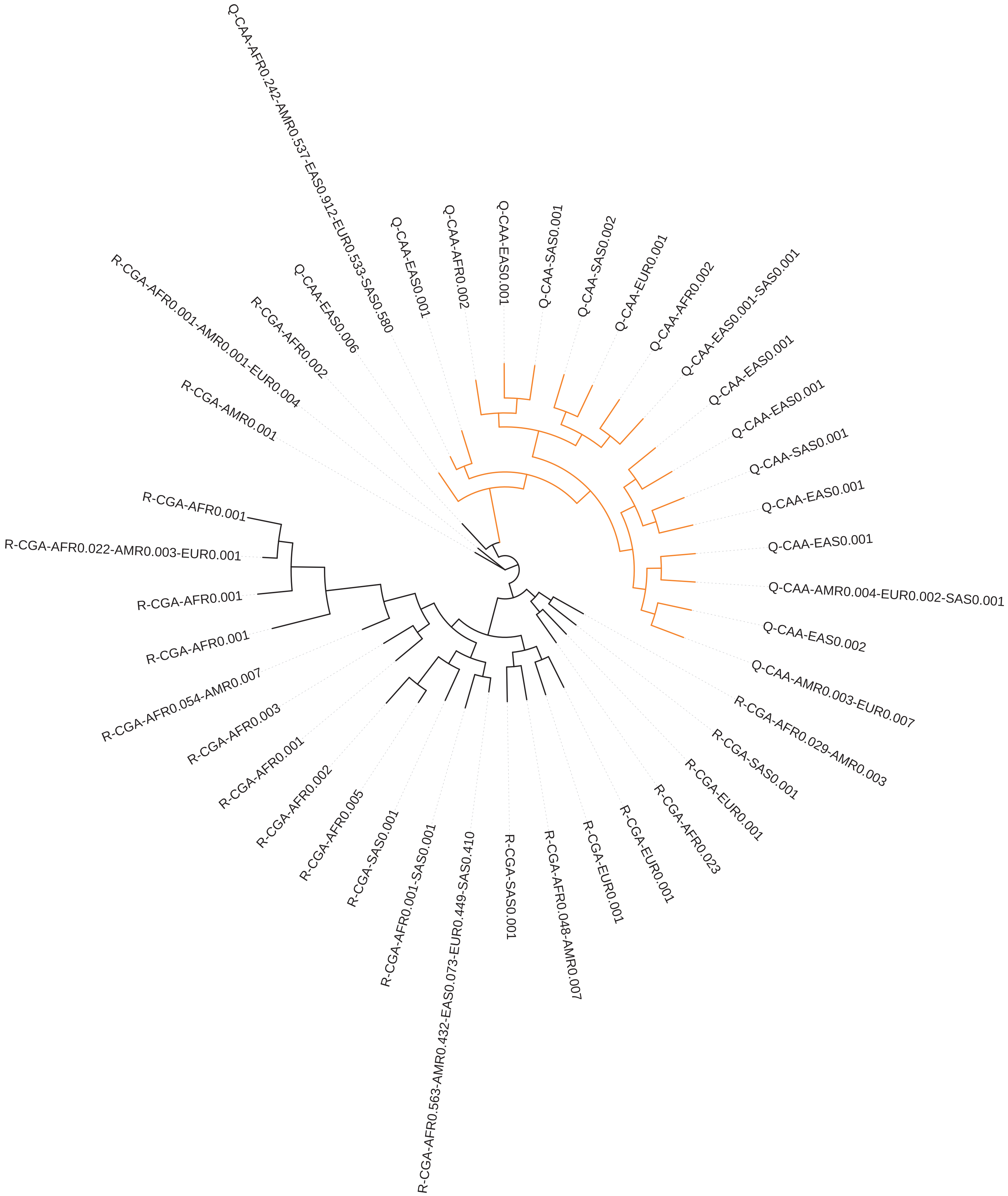
Q-CAA-AFR0.242-AMR0.537-EAS0.912-EUR0.533-SAS0.580
Q-CAA-EAS0.001
Q-CAA-AFR0.002
Q-CAA-EAS0.001
Q-CAA-SAS0.001
Q-CAA-SAS0.002
Q-CAA-EUR0.001
Q-CAA-AFR0.002
Q-CAA-EAS0.001-SAS0.001
Q-CAA-EAS0.001
Q-CAA-EAS0.001
Q-CAA-SAS0.001
Q-CAA-EAS0.001
Q-CAA-EAS0.001
Q-CAA-AMR0.004-EUR0.002-SAS0.001
Q-CAA-EAS0.002
Q-CAA-AMR0.003-EUR0.007
R-CGA-AFR0.029-AMR0.003
R-CGA-SAS0.001
R-CGA-EUR0.001
R-CGA-AFR0.023
R-CGA-EUR0.001
R-CGA-EUR0.001
R-CGA-AFR0.048-AMR0.007
R-CGA-SAS0.001
R-CGA-AFR0.563-AMR0.432-EAS0.073-EUR0.449-SAS0.410
R-CGA-AFR0.001-SAS0.001
R-CGA-SAS0.001
R-CGA-AFR0.005
R-CGA-AFR0.002
R-CGA-AFR0.001
R-CGA-AFR0.003
R-CGA-AFR0.054-AMR0.007
R-CGA-AFR0.001
R-CGA-AFR0.001
R-CGA-AFR0.022-AMR0.003-EUR0.001
R-CGA-AFR0.001
R-CGA-AMR0.001
R-CGA-AFR0.001-AMR0.001-EUR0.004
R-CGA-AFR0.002
Q-CAA-EAS0.006

Figure S3

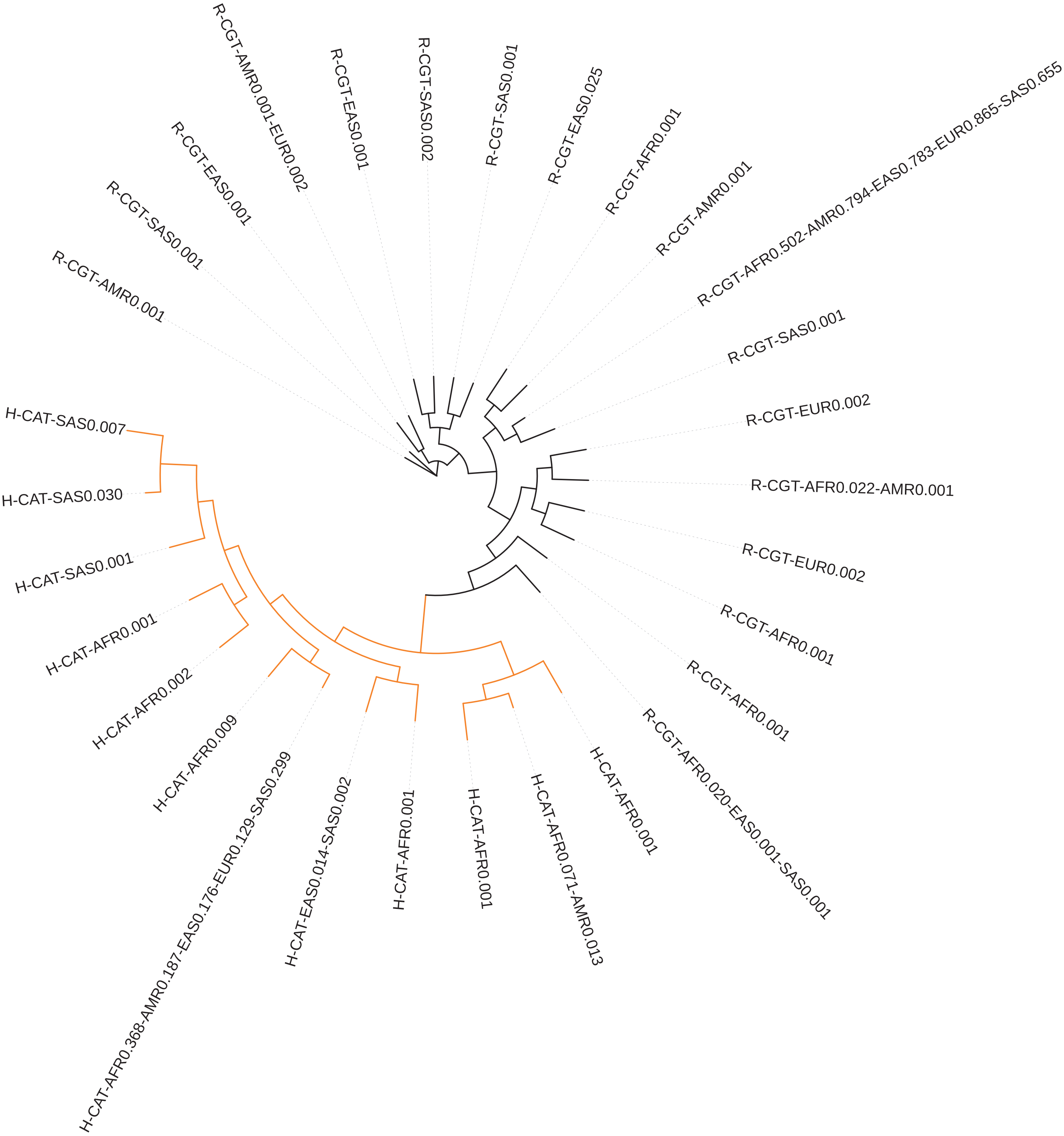

R-CGT-AMR0.001
R-CGT-SAS0.001
R-CGT-EAS0.001
R-CGT-AMR0.001-EUR0.002
R-CGT-EAS0.001
R-CGT-SAS0.002
R-CGT-SAS0.001
R-CGT-EAS0.025
R-CGT-AFR0.001
R-CGT-AMR0.001
R-CGT-AFR0.502-AMR0.794-EAS0.783-EUR0.865-SAS0.655
R-CGT-SAS0.001
R-CGT-EUR0.002
R-CGT-AFR0.022-AMR0.001
R-CGT-EUR0.002
R-CGT-AFR0.001
R-CGT-AFR0.001
R-CGT-AFR0.020-EAS0.001-SAS0.001
H-CAT-AFR0.001
H-CAT-AFR0.071-AMR0.013
H-CAT-AFR0.001
H-CAT-AFR0.001
H-CAT-EAS0.014-SAS0.002
H-CAT-AFR0.368-AMR0.187-EAS0.176-EUR0.129-SAS0.299
H-CAT-AFR0.009
H-CAT-AFR0.002
H-CAT-AFR0.001
H-CAT-SAS0.001
H-CAT-SAS0.030
H-CAT-SAS0.007

Figure S4

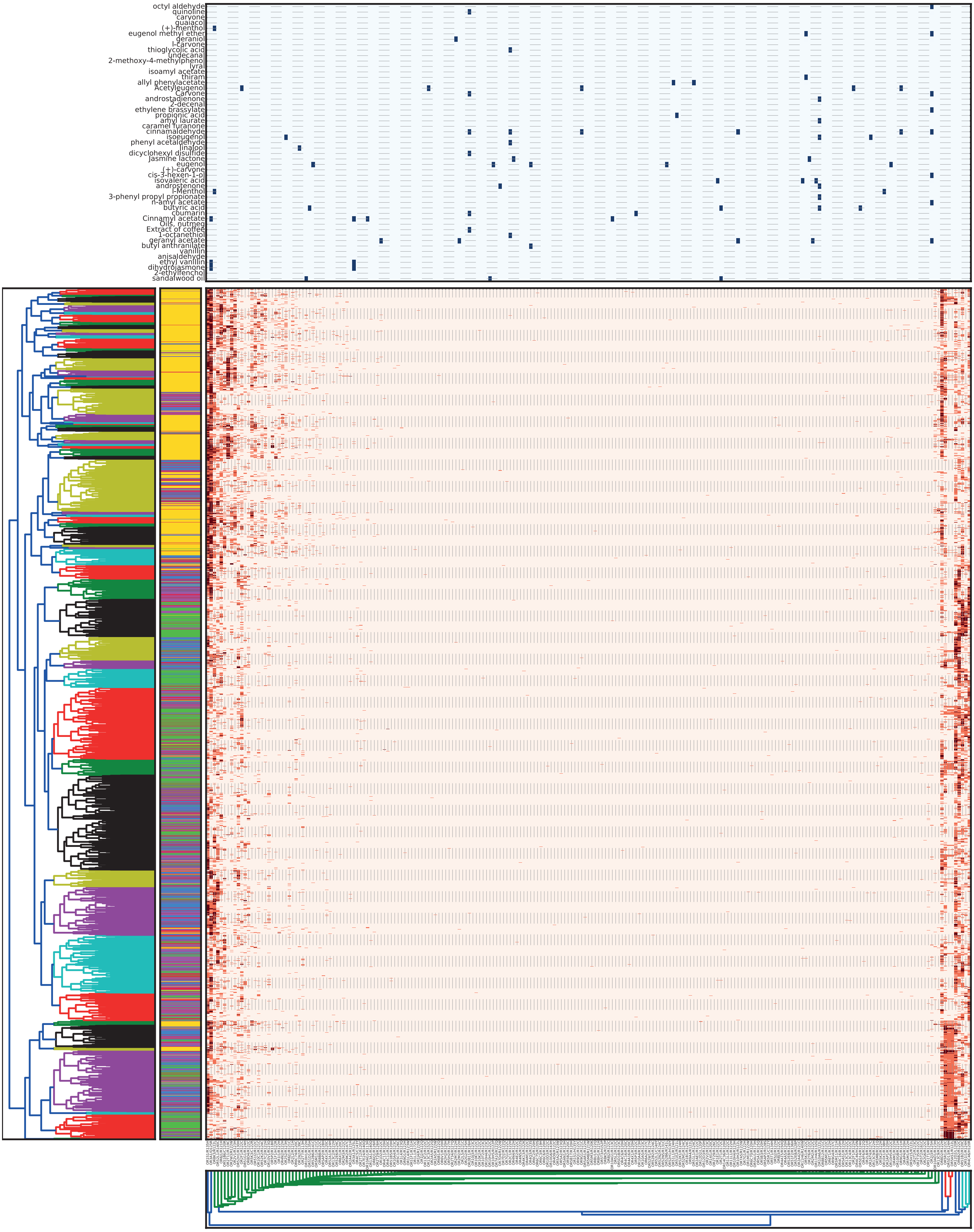
octyl aldehyde
quinoline
caryone
guaiacol
(+)-menthol
eugenol methyl ether
geraniol
l-carvone
thioglycolic acid
undecanal
2-methoxy-4-methylphenol
lyral
isoamyl acetate
thiram
allyl phenylacetate
Acetyleugenol
Carvone
androstadienone
2-decenal
ethylene brassylate
propionic acid
amyl laurate
caramel furanone
cinnamaldehyde
isoeugenol
phenyl acetaldehyde
linalool
dicyclohexyl disulfide
Jasmine lactone
eugenol
(+)-carvone
cis-3-hexen-1-ol
isovaleric acid
androstenone
l-Menthol
3-phenyl propyl propionate
n-amyl acetate
butyric acid
coumarin
Cinnamyl acetate
Oils, nutmeg
Extract of coffee
1-octanethiol
geranyl acetate
butyl anthranilate
vanillin
anisaldehyde
ethyl vanillin
dihydrojasmone
2-ethylfenchol
sandalwood oil

Figure S5

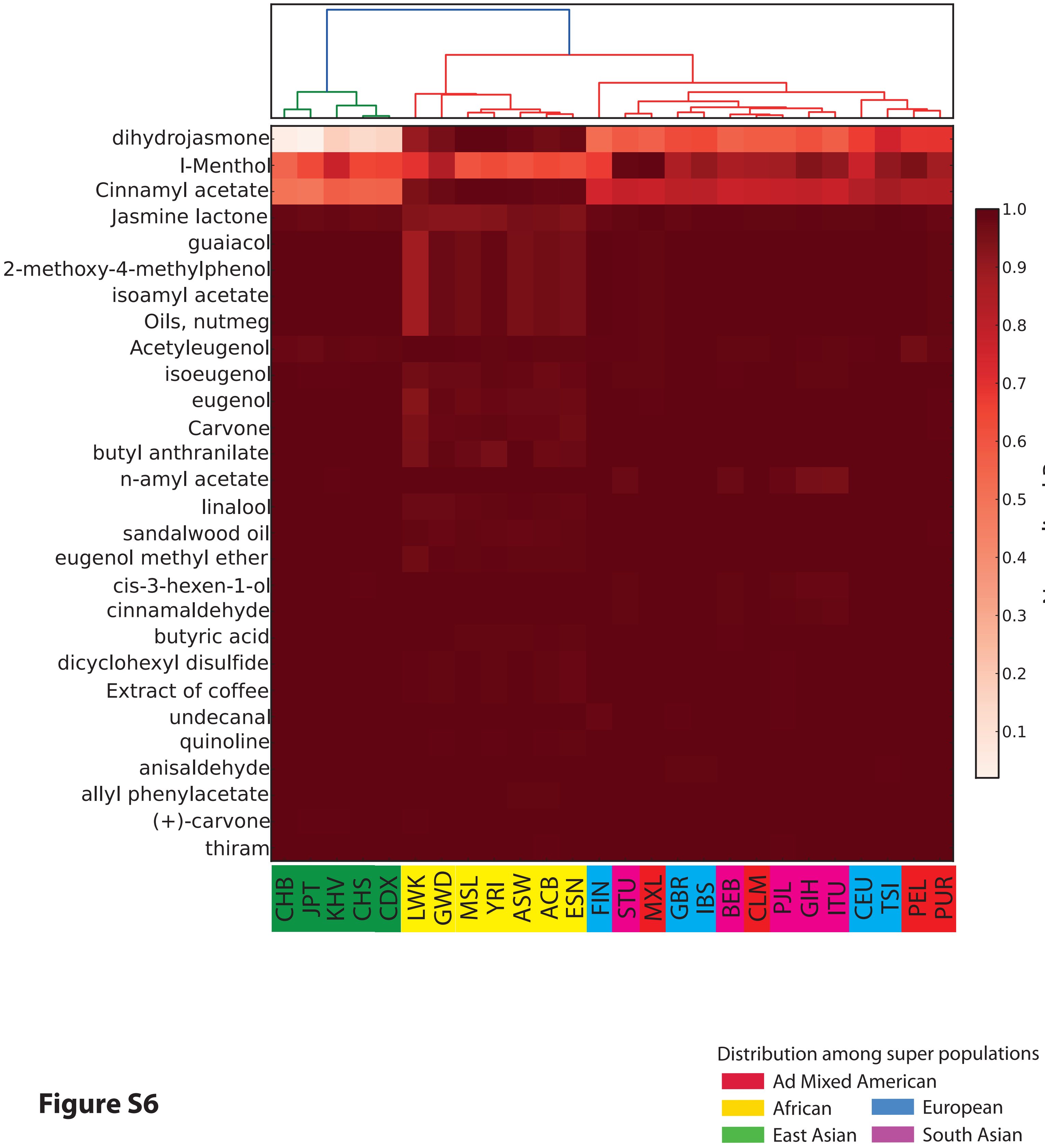
dihydrojasmone
l-Menthol
Cinnamyl acetate
Jasmine lactone
guaiacol
2-methoxy-4-methylphenol
isoamyl acetate
Oils, nutmeg
Acetyleugenol
isoeugenol
eugenol
Carvone
butyl anthranilate
n-amyl acetate
linalool
sandalwood oil
eugenol methyl ether
cis-3-hexen-1-ol
cinnamaldehyde
butyric acid
dicyclohexyl disulfide
Extract of coffee
undecanal
quinoline
anisaldehyde
allyl phenylacetate
(+)-carvone
thiram
CHB JPT KHV CHS CDX LWK GWD MSL YRI ASW ACB ESN FIN STU MXL GBR IBS BEB CLM PJL GIH ITU CEU TSI PEL PUR
1.0
0.9
0.8
0.7
0.6
0.5
0.4
0.3
0.2
0.1
Normalized Response
Distribution among super populations
Ad Mixed American
African
East Asian
European
South Asian

Figure S6

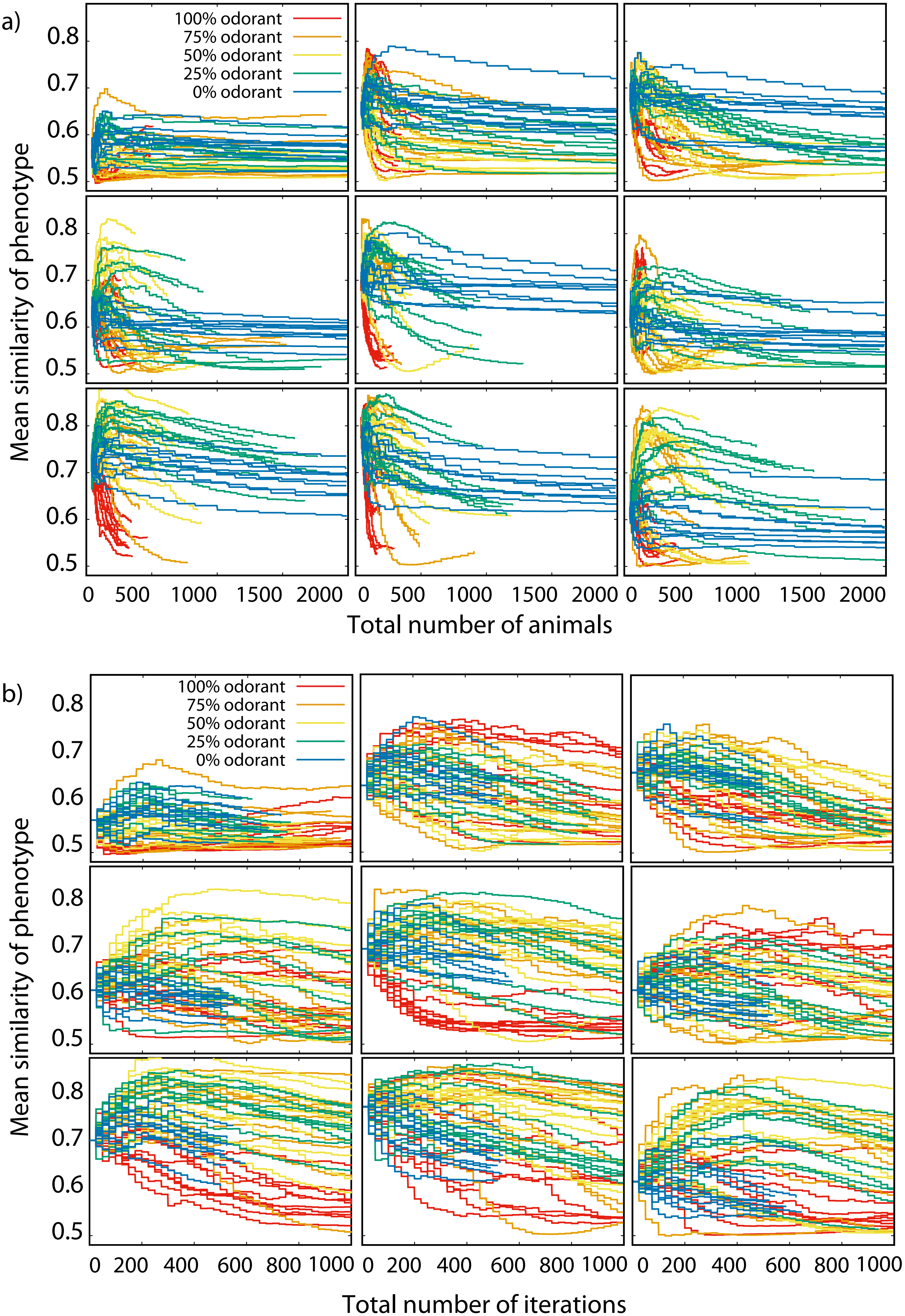
a)
100% odorant
75% odorant
50% odorant
25% odorant
0% odorant
Mean similarity of phenotype
0.8
0.7
0.6
0.5
0 500 1000 1500 2000
Total number of animals
b)
Total number of iterations
0 200 400 600 800 1000

Figure S7
